# Monitoring the Starvation–Survival Response of *Edwardsiella piscicida* and *E. tarda* in Freshwater Microcosms, at Various Temperatures

**DOI:** 10.3390/microorganisms10051043

**Published:** 2022-05-18

**Authors:** Consuelo Esteve, Elena Alcaide

**Affiliations:** Departamento de Microbiología y Ecología, Universitat de València, Campus de Ciencias Burjassot-Paterna, E-46100 Valencia, Spain; elena.alcaide@uv.es

**Keywords:** *E. piscicida*, *E. tarda*, starvation survival, VBNC state, biofilm, retention of virulence

## Abstract

*Edwardsiella piscicida* is an important fish pathogen responsible for economic losses in global aquaculture, and *E. tarda* is also a human zoonotic pathogen. In this study, the survival of *E. piscicida* and *E. tarda* strains kept in filtered and sterilized lake water microcosms was investigated during a 20-week period at 7 °C, 15 °C and 25 °C, as well as its pathogenicity retention during a starvation period. *E. tarda* V43.2 stayed culturable for 6 weeks at 7 °C, 9 weeks at 25 °C and 12 weeks at 15 °C. Both *E. piscicida* strains (V12.1 and V57.2) stayed culturable even longer, for at least 12 weeks at 7 °C, 15 °C and 25 °C under the same starvation conditions. After *Edwardsiella* cells entered into the VBNC state, some became shorter and ”rounded up,” but others aggregated and retained a short rod shape. Aggregates of *Edwardsiella* cells were common throughout the VBNC period, and a well-formed biofilm was observed for all tested strains at the end of the experiment. The growth capacity of VBNC cells was restored by cultivating microcosm water samples in LB broth at 28 °C. Resuscitated *E. piscicida* cells were as virulent for the European eel as the controls. Natural waters can be a reservoir for *Edwardsiella*, and its underestimation in environmental samples poses a risk to public health and aquaculture.

## 1. Introduction

The genus *Edwardsiella* includes enteric gram-negative bacteria of the *Hafniaceae* family [1] that are causative agents of disease in homeothermic and poikilothermic animals [2,3]. The species *E. tarda*, *E. ichtaluri*, *E. piscicida* and *E. anguillarum* are related to the systemic disease Edwardsiellosis, which has been associated with mass mortality in more than 20 species of fish reared in freshwater and marine-supplied aquaculture systems worldwide [4,5].

The search for *Edwardsiella* reservoirs in wild aquatic vertebrates and natural surface waters has primarily been a public health issue [6,7] because *E. tarda* cause water- and foodborne infection in humans [2]. To date, the *Edwardsiella* have been effectively isolated from diseased aquatic vertebrates [8,9,10] and, to a lesser extent, from “healthy carriers” [6,10,11]. In addition, *E. tarda* have been isolated from natural surface freshwater on a few occasions [8,10]. Regarding *E. piscicida*, the only data on its presence in nature describe its isolation from wild eels (*Anguilla anguilla*), but scarcely from lagoon freshwater, with all samples being collected at the L’Albufera Lake (Mediterranean Spain) over a 12-year period [10,12]. Since Edwardsiellosis disease arises within populations in warm seasons [10,13,14], its continuous incidence in the fishery over a long time period is an intriguing problem [10,12].

The classic experiments to evaluate the role of water as a reservoir of many bacterial pathogens and, therefore, to study their survival response to lack of nutrients, have been carried out using microcosms of filtered and sterilized water [15,16,17,18]. Du et al. [15] studied the survival response of *E. tarda* strain CW7 in the microcosms of filtered sterilized seawater (FSSW) maintained at 4 °C for 42 days. They reported no more than 28 days of cultivability for strain *E. tarda* CW7 at 4 °C under nutrient deprivation, after which it entered the viable but non-cultivable (VBNC) state [15]. Many species of bacteria enter the VBNC state when exposed to stressful conditions such as starvation and low temperatures [15,16,17,18], and this bacterial adaptability could pose a risk to animal and human health. The risks emerge from the fact that pathogenic bacteria can be avirulent in the VBNC state but regain virulence after resuscitation into culturable cells under suitable conditions [16,17,18]. This was the case for resuscitate cells of *E. tarda* CW7 that were as virulent for fish as the original strain [15]. For now, the survival response to starvation of *E. piscicida* is unknown because no research has been conducted on this topic.

To better understand the environmental epidemiology of these pathogens, we studied the starvation response of two *E. piscicida* and one *E. tarda* strains in filtered and sterilized lake water (FSLW) microcosms maintained at cold (7 °C), temperate (15 °C) and warm (25 °C) temperatures. This study represents the first assessment of the response of *E. piscicida* and *E. tarda* to nutrient deprivation in water over a period of 20 weeks (i.e., 140 days), showing its ability to survive and remain pathogenic in freshwater at several temperatures (7 °C, 15 °C and 25 °C), as well as its ability to form biofilms under environmental stress.

## 2. Materials and Methods

### 2.1. Bacterial Strains and Culture Conditions

Three *Edwardsiella* strains were used in the study: *E. piscicida* V12.1 isolated from kidney of diseased European eel [19], *E. piscicida* V57.2 isolated from intestine of healthy European eel [10] and *E. tarda* V43.2 isolated from lagoon freshwater [10]. These strains were previously identified by biochemical traits as well as 16S rRNA sequencing [10]. Unless otherwise stated, cells were grown in Luria Bertani (LB, Pronadisa, Alcobendas, Spain) broth and agar (LBA, Pronadisa) at 28 °C for 24 h.

### 2.2. Preparation of the Filtered and Sterilized Lake Water (FSLW) and Inoculation of Microcosms

Natural fresh water collected at a depth of 20 cm from Lake L’Albufera (Mediterranean Spain) was used. In the laboratory, lagoon freshwater was filtered through membrane filters (Millipore, 0.45 µm pore size) to remove all particles, placed in 125 mL Erlenmeyer flasks with a stopper (each containing 75 mL of water) and finally autoclaved at 121 °C for 20 min. The physicochemical parameters of the FSLW were BOD_5_ = 8 mg O_2_/L regarding of dissolved organic matter; pH = 8.2; salinity = 0.6 g/L.

Bacteria were grown in LBA at 28 °C, and harvested and washed twice in quarter-strength Ringer’s solution (Oxoid, Hampshire, UK). Then, *Edwardsiella* cells pellet was re-suspended into FSLW, and added into the FSLW microcosms in a final concentration of 10^7^–10^8^ CFU/mL. The seeded microcosms were incubated without shaking (static microcosms) at 7 °C, 15 °C or 25 °C and maintained in the dark for 20 weeks. Experiments were performed in triplicate (three simultaneous microcosms) for each *Edwardsiella* strain and temperature condition. Temperature values were chosen according to water temperature records previously measured at the L’Albufera Lake [10].

### 2.3. Cell Counts, Viability Assessment and Staining Methods

Cell counts were determined immediately after microcosm inoculation and then at a weekly or biweekly interval over an 18-week period. A last counting was performed at the 20-week mark. Microcosms were gently shaken prior to take the sample for counting.

Direct counts of viable and total bacterial cells were determined in 7 °C and 25 °C microcosm samples by staining with Syto 9 and propidium iodide (PI) (Baclight LIVE/DEAD kit, Molecular Probes Inc., Eugene, OR, USA) for 20 min. The stained samples were added to 5 mL of sterile Scharlau water and filtered on 0.22 µm black Nucleopore filters (GTPB filter type, Millipore, Burlington, MA, USA). The prepared filters were observed using epifluorescence microscopy (Nikon ECLIPSE E800, Tokyo, Japan) in 20 to 30 randomly selected fields for a total count, and the number of total (reed and green cells) and viable (green cells) bacteria was calculated, respectively. Morphological changes were annotated and photographed.

The culturable cell counts were determined in 7 °C, 15 °C and 25 °C-microcosms by seeding 3 plates of Luria-Bertani Agar (LBA, Pronadisa) per each water microcosm dilution, and then incubated for 24–48 h at 28 °C. The dilution chose for counting was always between 20 and 200 colonies on LBA plates. The number of colony-forming units (CFU) per mL was calculated from the number of colonies, the volume of the inoculum used (0.1 mL) and the dilution factor. Finally, the CFU/mL for each *Edwardsiella* strain and temperature treatment was expressed as (average ± standard deviation) from the results obtained in the three simultaneous microcosms. Colonies counted in these LBA cultures were sub-cultured onto Salmonella Shigella agar (SSA, Oxoid) to check that they showed the typical *Edwardsiella* morphology (i.e., transparent colonies with a black center as it is a non-lactose-fermenting and H_2_S-producing organism). Once the plate counts were negative for *Edwardsiella* (below the detection limit of 1 CFU per 0.1 mL), samples of 1 mL were taken directly from the microcosm and plated on LBA plates (3 plates) that were incubated at 28 °C for 48 h. At this point, the cells were considered to be in the viable non-culturable state (VBNC) if no growth was achieved.

### 2.4. Resuscitation of VBNC Cells

Microcosm samples of 7 °C, 15 °C and 25 °C that did not grow in LBA plates were taken at 1, 2, 3, 4, 5 and 6 weeks after entering the VBNC state in order to restore the culture capacity of the strain, according to the method described by the authors of [20].

Briefly, aliquots of 1 mL samples of microcosm water were diluted (from 10^−1^ to 10^−7^) in 9 mL tubes of Luria Bertani broth (LBB, Pronadisa) to reduce the probability (P) of any culturable cell down to <0.0000001 CFU/mL. These broth cultures were incubated at 28 °C for 2–4 days with shaking. Those with positive growth were seeded on Salmonella Shigella agar (SSA, Oxoid), and the plates were incubated at 28 °C for 24–48 h. Typical *Edwardsiella* colonies in SSA were sub-cultured on LBA to obtain pure cultures that were identified [10].

### 2.5. Virulence of the Original Edwardsiella Strains and of Its Resuscitated Cultures for European Eel

Animal experiments were carried out in accordance with the EU Directive [21]. Twelve juvenile eels of 10–15 g were used for the challenge dose. All eels were supplied by the “Piscifactoria de Tuéjar (Azud de Tuéjar. Generalitat Valenciana)” and certified to be disease-free.

The virulence of each original *Edwardsiella* isolate and its resuscitated strains was assessed by an intraperitoneal (IP) injection challenge at a unique dose of 10^7.3^ to 10^5.8^ CFU/fish as previously described [10]. A control group was used for each trial in which the fish were injected IP with 0.1 mL of sterile saline. The mortalities were recorded daily for a 5-day period and were only considered if the challenged strain was recovered as pure cultures from the internal organs. In all experiments, the percentage of accumulative mortality was determined, the CFUs of the bacterial suspensions were obtained on LBA at 28 °C by the “drop-plate method” [22] and the survivors were sacrificed by 20 min inhalation of anesthesia with benzocaine at 200 mg/L (wt./vol.).

### 2.6. Data Analysis and Statistics

A one-way analysis of variance (ANOVA) with Tukey’s method was used to assess the statistical significance of the differences observed in VBC counts as a function of incubation temperature. For each strain, data were analyzed if VBC counts were obtained from all temperatures.

## 3. Results

### 3.1. Culturability of Edwardsiella Maintained under Starvation Conditions at Various Temperatures

**Figure 1:**
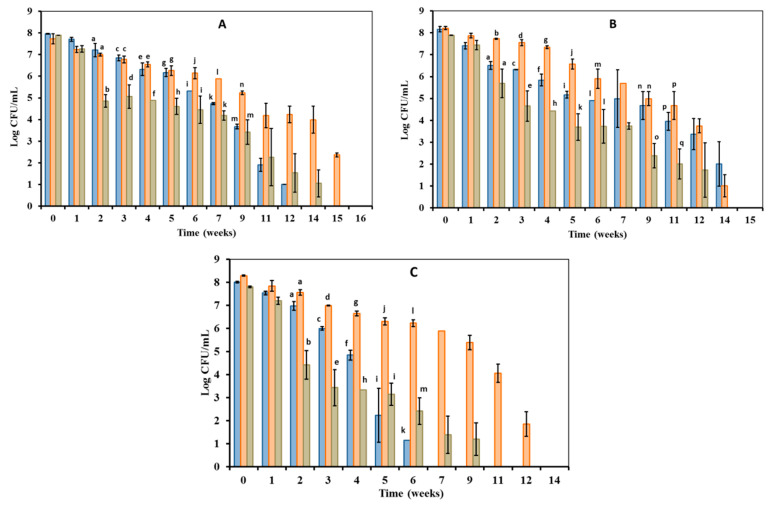
Culturable cell (VBC) numbers of *Edwardsiella* maintained under starvation conditions in FSLW microcosms at 7 °C (blue bars), 15 °C (orange bars) and 25 °C (green bars): (**A**) *E. piscicida* V12.1; (**B**) *E. piscicida* V57.2; (**C**) *E. tarda* V43.2. For each strain, values with different letters indicate significant differences at *p* < 0.05.

Culturable cell (VBC) numbers in *Edwardsiella* populations were around 10^8^ CFU/mL at the beginning of the experiment (Figure 1), which lasted 20 weeks. In general, we found differences between *E. piscicida* (Figure 1A,B) and *E. tarda* (Figure 1C) regarding their culturability behavior when they were kept under starvation conditions.

The VBC numbers of *E. piscicida* kept at 7 °C decreased from 10^8^ to 10^5^ CFU/mL after 7 weeks of incubation (Figure 1A,B), while that of *E. tarda* were of 0 CFU/mL (Figure 1C). The two *E. piscicida* strains entered into viable non-culturable state (VBNC: 0 CFU/mL) significantly later than the *E. tarda* strain because they stayed culturable onto LBA plates until 12 weeks (Figure 1A) or 14 weeks (Figure 1B) of incubation.

All *Edwardsiella* kept at 15 °C under starvation conditions maintained VBC counts above 10^5^ CFU/mL until at least 9 weeks of incubation (Figure 1). Then, the two *E. piscicida* strains stayed culturable for an additional 5 or 6 weeks, entering into the VBNC state (0 CFU/mL) after 15 weeks (Figure 1A) or week 14 (Figure 1B) of incubation. The *E. tarda* strain entered into VBNC state earlier than E. piscida ones because 0 CFU/mL counts were already recorded after the 12 weeks of incubation (Figure 1C).

All microcosms kept at 25 °C under starvation conditions showed an acute decreasing in VBC *Edwardsiella* numbers of at least 3-log units during the second week of incubation (Figure 1). After, the VBC numbers of the two E. piscicida strains slowly decreased along study period, and stayed culturable even at levels of around 10^3^ CFU/mL at 9–11 weeks of incubation (Figure 1A,B). Finally, E. piscicida strains entered into the VBNC state after 14 weeks (Figure 1A) or 12 weeks (Figure 1B) of incubation. In contrast, VBC numbers of the *E. tarda* strain V43.2 decreased rapidly from the second week to the end of the experiment, and the strain entered into the VBNC (0 CFU/mL) after 9 weeks of incubation (Figure 1C).

Furthermore, the observed differences in VBC counts as a function of microcosm incubation temperature were statistically significant for *Edwardsiella* strains maintained under the same starvation conditions (Figure 1). Regadless of their taxonomic assignment, all *Edwardsiella* showed the highest numbers of VBC counts in microcosms at 15 °C (Figure 1). *E. piscicida* strains frequently showed significantly higher VBC counts in microcosms at 7 °C than in microcosms at 25 °C throughout the experimental period (Figure 1A,B). In contrast, *E. tarda* seemed to maintain more stable VBC numbers in microcosm at 25 °C than in microcosms at 7 °C (Figure 1C).

### 3.2. The VBNC State of Edwardsiella Maintained under Starvation Conditions at 7 °C and 25 °C

#### 3.2.1. Direct Counts of *Edwardsiella* Cells during Culturable and VBNC Period

**Figure 2:**
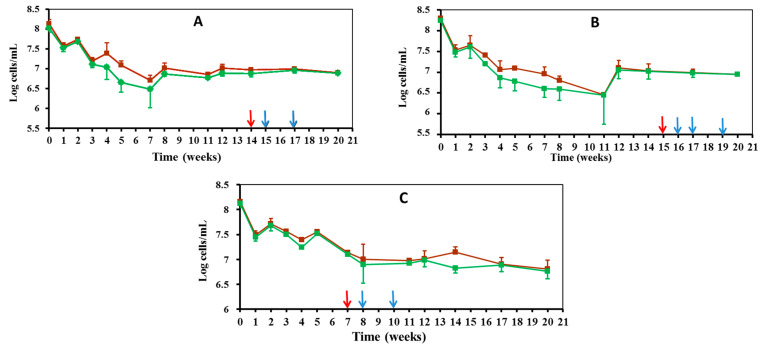
Direct counts of total (brown line) and viable (green line) *Edwardsiella* cells maintained under starvation conditions in FSLW microcosms at 7 °C: (**A**) *E. piscicida* V12.1; (**B**) *E. piscicida* V57.2; (**C**) *E. tarda* V43.2. Red arrow indicates the beginning of the VBNC period. Blue arrows indicate the timing of resuscitation experiments of VBNC *Edwardsiella* cells.

**Figure 3:**
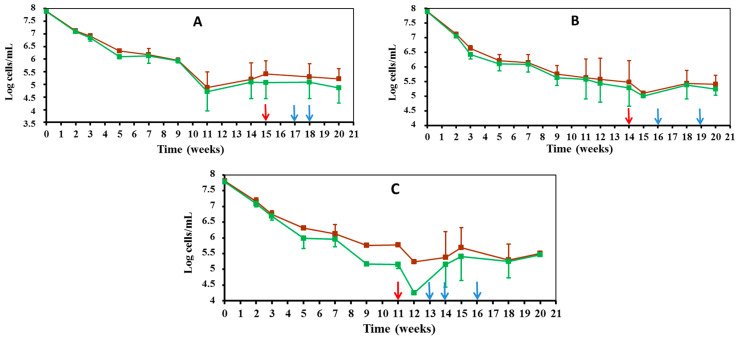
Direct counts of total (brown line) and viable (green line) *Edwardsiella* cells maintained under starvation conditions in FSLW microcosms at 25 °C: (**A**) *E. piscicida* V12.1; (**B**) *E. piscicida* V57.2; (**C**) *E. tarda* V43.2. Red arrow indicates the beginning of the VBNC period. Blue arrows indicate the timing of resuscitation experiments of VBNC *Edwardsiella* cells.

Throughout the period in which the tested strains decreased in cultivability (Figure 1), the initial size of direct *Edwardsiella* counts also showed a marked decrease, regardless of the strain taxonomic assignment and the incubation temperature (7 °C: Figure 2, 25 °C: Figure 3).

Such a numerical reduction could show a rapid initial collective death rate in the *Edwardsiella* population, which would then benefit viable cells, as they can recycle the biomass of dead ones [23]. In fact, direct *Edwardsiella* cell counts remained stable throughout the VBNC period, the beginning of which is marked on the graphs with a red arrow (Figure 2 and Figure 3). The direct total and viable *Edwardsiella* cell counts during the VBNC period and until the end of the experiment remained around 10^7^ cells/mL in the 7 °C microcosms (Figure 2). These counts were at lower levels of 10^6^–10^5^ cells/mL in the 25 °C microcosms (Figure 3).

#### 3.2.2. Morphological Changes Observed in *Edwardsiella* Cells during Starvation-Survival Response

**Figure 4:**
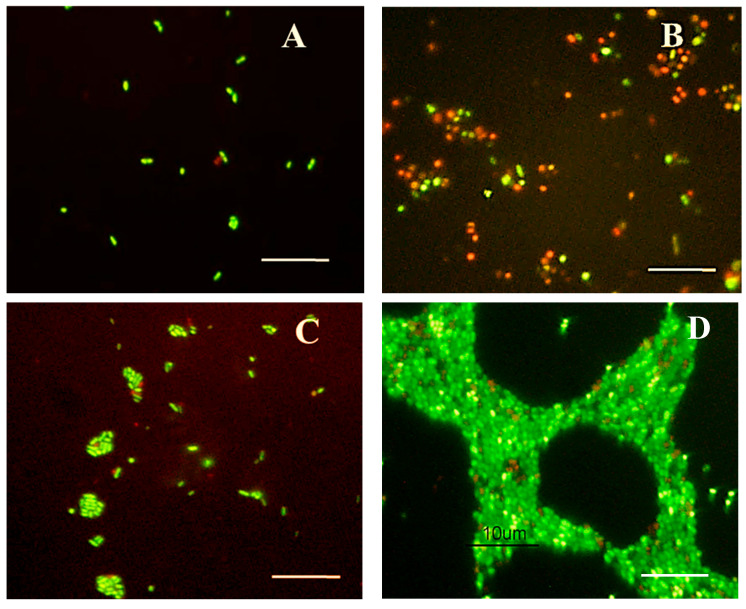
Morphological changes of *Edwardsiella* cells maintained under starvation conditions. (**A**) Viable bacillary cells in 2-day-old microcosms (10 µL were used for LIVE/DEAD staining); (**B**) Viable and non-viable rounded cells, and viable bacillary cells in 4-week-old microcosm; (**C**) Viable and non-viable rounded cells, and aggregates of viable bacillary cells observed in 10-week-old microcosm; (**D**) Biofilm composed primarily of short rod-shaped viable cells in 20-week-old microcosm. Bars = 10 µm.

Samples (100 µL) of microcosm water were stained with Syto 9 and propidium iodide (LIVE/DEAD staining) to reveal viable/live (green-stained) and dead (red-stained) *Edwardsiella* cells. Morphological changes of *Edwardsiella* cells maintained under starvation conditions for 20 weeks at 7 °C or 25 °C were observed under an epifluorescence microscopy (Figure 4).

The two strains of *E. piscicida* and the strain of E. *tarda* showed similar morphological changes upon entering into the VBNC state. *Edwardsiella* cells were single or paired rods (Figure 4A) that progressively changed over their cultivable period to rounded viable cells (Figure 4B), rounded non-viable cells (Figure 4B) or rod-shaped viable cells in aggregates (Figure 4C).

We observed that these three morphological types could coexist in the microcosms at the same time, at the beginning of the VBNC period. Finally, a biofilm, primarily including short rod-shaped viable cells and some non-viable rounded cells, was observed for all tested strains at the end of the experiment (Figure 4D). Those viable cells inside the biofilms presented a bright green color or a darker green color by Syto9. We did not find that LIVE/DEAD staining underestimated the viability of these adherent cells as others described [24].

#### 3.2.3. Resuscitation of VBNC *Edwardsiella* Cells and Its Virulence for European Eel

Samples were taken from 7 °C, 15 °C and 25 °C microcosms that did not grow on LBA plates (VBNC: 0 CFU/mL, Figure 1) after 1, 2, 3, 4, and 5 weeks of entering the VBNC state (Figure 2 and Figure 3), with the purpose of restoring the culture capacity of the strain. The growth capacity of VBNC cells was restored in both *E. piscicida* and *E. tarda* strains using a rich general medium (i.e., LB broth) to culture the microcosm water samples. Furthermore, an increase in temperature could have positively affected *Edwardsiella* cultivability in the case of microcosms at 7 °C and 15 °C, because all samples were incubated at 28 °C. The “resuscitation window” [25] was different depending on the microcosm temperature. The longest resuscitation period was observed for the microcosms kept at 15 °C because growing *Edwardsiella* cells recovered 2–4 weeks after entering the VBNC state (Table 1). A narrower resuscitation window was observed for the VBNC cells from those microcosms maintained at 7 °C and 25 °C. The recovery of growing *Edwardsiella* after entering the VBNC state was limited to 1 week for microcosms maintained at 7 °C and 2 weeks for those maintained at 25 °C (Table 1).

The resuscitated *E. piscicida* strains were virulent for eels by intraperitoneal injection (IP) challenge at doses of 10^6^ CFU/g of fish. They caused an accumulative mortality of 67–100% among challenged fish (Table 1). Dead and moribund fish showed pathological signs previously observed in sampled wild eels suffering from edwardsiellosis [10]. Although these strains were generally as pathogenic as the original strain, we observed that the longest lapse of time after the induction of the VBNC state could have contributed to reduction of the pathogenicity of one out of the five resuscitated *E. piscicida* tested (Table 1). The original *E. tarda* V43.2 strain, as well as its resuscitated strains, yielded 0% of accumulative mortality among challenged fish; therefore, they were avirulent for European eel (Table 1) in contrast to *E. tarda* CW7 [15]. The *E. tarda* CW7 was recovered from diseased fish [15]. Therefore, it could belong to another *Edwardsiella* species based on the current taxonomic viewpoint [5]. In fact, the genetic divergence observed for fish isolates of *E. tarda* [26,27] was the first evidence that later led to the proposals of *E. piscicida* and *E. anguillarum* [28,29]. The fish pathogenic *Edwardsiella* (i.e., *E. ichtaluri*, *E. piscicida* and *E. anguillarum*) constitute a clearly segregated branch of *E. tarda* and *E. hoshinae* in the *Edwardsiella* phylogenetic tree [29,30]. Thus, strains previously defined as “fish pathogenic *E. tarda*” belong to the species *E. piscicida* or *E. anguillarum*, whereas strains of *E. tarda* that are non-pathogenic for fish are consistent with the species *E. tarda*, as currently defined [5,30].

## 4. Discussion

A recent paper introduced that *Edwardsiella* exist in aquatic environments either as individual planktonic cells or in communal biofilms [31]. However, biofilm production by *Edwardsiella* has been only related to its life as a pathogen for vertebrate animals [32,33]. Moreover, *E. piscicida* and *E. tarda* have hardly been isolated from surface water in natural environments, and work on this subject is limited. White et al. [8] recovered *Edwardsiella* from 13 out of 108 water samples (12%), and Esteve and Alcaide [10] recovered *Edwardsiella* from 2 out of 27 water samples (7%). Some authors have highlighted the role of the intestine as a gateway for the *Edwardsiella* since they can cross intestinal mucosa, such as other enteric pathogens ingested through water or food [2,34]. Although the presence of *Edwardsiella* in the intestines of healthy wild fish has been reported [6,10,11], the role of water as a reservoir for these bacteria is still unknown. Specifically, it is unknown whether the *Edwardsiella* are capable of surviving in such an oligotrophic environment even after their exposure to different temperature conditions.

Du et al. [15] reported no more than 28 days of culturability for the strain *E. tarda* CW7 maintained in filtered and sterilized seawater (FSSW) at 4 °C under nutrient starvation. In contrast, our strain, *E. tarda* V43.2, remained cultivable for 42 days in filtered sterilized freshwater (FSLW) microcosms at 7 °C, and slightly longer at warm (25 °C) temperature, similar to that observed for other bacterial pathogens of humans and fish [16,17]. The two strains of *E. piscicida* tested (V12.1 and V57.2) remained cultivable even longer, for 84 days at 7 and 25 °C, and around 100 days at 15 °C, under the same conditions. In addition, the highest number of viable cultivable (VBC) cells was found in the microcosms kept at 15 °C, showing a statistically significant difference. Unlike other fish pathogens, *E. piscicida* did not prefer low temperatures [18] or warm temperatures [16,17]. After the tested *E. piscicida* and *E. tarda* strains lost the ability to grow on Luria-Bertani agar, however, all FSLW microcosms had live *Edwardsiella* cells (i.e., viable non-cultivable (VBNC) cells) until the end of the experiment (140 days). These results show for the first time that the species *E. piscicida* exists in the VBNC state in response to nutrient deprivation.

The entry into the VBNC state of *E. piscicida* V12.1 and V57.2 and *E. tarda* V43.2 caused a reduction in the size of some cells until they became coccoid cells, as described for other gram-negative pathogens [15,16,17,18]. This rounding and cell size reduction is well known to increase the cell surface area to volume ratio, which is an advantage in oligotrophic environments [35]. Furthermore, our starved *Edwardsiella* strains exhibited various cell types at the beginning of the VBNC period, as observed by epifluorescence microscopy, which showed cylindrical rods, shorter rod-shape cells and spherical-shaped cells similar to that described for *Escherichia coli* K-12 [36]. The coexistence of different morphological types within starving bacterial populations have been linked in *Vibrio* species to starvation-induced phenotypic diversification [37,38]. Rod-shaped *Edwardsiella* cells forming aggregates were also observed in the VBNC period. In fact, our results show for the first time that starved populations of *E. piscicida* and *E. tarda* produce a biofilm, primarily including short rod-shaped viable cells and some non-viable rounded cells, after prolonged incubation in the VBNC period. Such loss of motility, as well as the increase in adhesion properties, including biofilm production, has been reported for other intestinal pathogens maintained in lake water microcosms, suggesting that biofilm might has a role in their survival in natural waters [39].

It is important to note that bacteria that enter the VBNC state may become culturable again. Thus, this state may be reversible in the presence of a suitable stimulus (i.e., rich medium, temperature upshift, presence of host cells) [35]. Of the bacterial pathogens that can enter the VBNC state (52 reported species), resuscitation has been reported in only half [35]. The risk emerges from the fact that many of them are avirulent in the VBNC state but regain virulence after resuscitation into culturable cells [15,35]. Similarly, we found that *E. piscicida* cells resuscitated from the VBNC state were as virulent to European eel as the original strain. Such behavior was limited to the “resuscitation window” involving younger *E. piscicida* VBNC cells, because a reduction in resuscitable cells was observed over time, consistent with other reports [40]. Specifically, the length of the “resuscitation window” observed for our *Edwardsiella* strains ranged from 1 to 2 weeks after entering into the VBNC state. This corresponded to the 112–119 days after the *E. piscicida* strains began the nutrient depletion conditions in FSLW microcosms. This work represents the first description of the starvation response of *E. piscicida*, evidencing its survival and pathogenic potential in freshwater at a wide range of temperatures. Moreover, it also delves into the starvation response of *E. tarda* compared to what was previously reported [15]. These findings suggest that natural freshwater may be a reservoir for *Edwardsiella* because it can adapt to survive in such an oligotrophic environment. Therefore, its low recovery from these samples could be explained by the use of culture media that are not selective enough to reduce the growth of accompanying bacteria (i.e., *Enterobacter* spp., *Citrobacter* spp., *Aeromonas* spp., *Plesiomonas shigelloides* and *Hafnia alvei*) [7,10]. Even the effect of microbial interactions with other bacteria or phages should not be ruled out as relevant stress factors for the survival of *Edwardsiella* in natural waters. Future research will focus on the effect of accompanying bacteria on the survival of *Edwardsiella* in water.

## 5. Conclusions

*Edwardsiella piscicida* and *E. tarda* can survive in freshwater under nutrient deprivation for long time periods, evidencing its starvation response and pathogenic potential at a wide range of temperatures (7–25 °C). Specifically, a temperate temperature (15 °C) favors the persistence of *E. piscicida* in the aquatic environment in a culturable and pathogenic state. However, lower and higher temperatures in other periods of the year would induce a more rapid development of the VBNC response, with an enhanced resistance to environmental stress due to biofilm formation. These VBNC cells can recover their pathogenicity under appropriate conditions according to a resuscitation window that is also temperature-dependent. This knowledge provides new and valuable information on the biology of this pathogen; therefore, it leads us to a better understanding of the environmental epidemiology of diseases caused by *Edwardsiella*.

## Figures and Tables

**Table 1:** Mortality of juvenile eels (10–15 g) injected with *Edwardsiella piscicida* and *E. tarda* strains.

Strains	IP Challenges Dose (CFU/g of Fish)	5 Days-Accumulative Mortality
*Edwardsiella piscicida*		
V12.1 original	1.20 × 10^6^	83%
V12.1_7 °C_R_1 w ^1^	0.64 × 10^6^	100%
V12.1_15 °C_R_2 w	1.00 × 10^6^	80%
V12.1_25 °C_R_2 w	1.00 × 10^6^	67%
*Edwardsiella piscicida*		
V57.2 original	2.30 × 10^5^	80%
V57.2_7 °C_R_1 w	1.42 × 10^6^	100%
V57.2_15 °C_R_2 w	0.70 × 10^6^	80%
*Edwardsiella tarda*		
V43.2 original	1.10 × 10^7^	0%
V43.2_7 °C_R_1 w	2.16 × 10^7^	0%
V43.2_15 °C_R_1 w	2.42 × 10^7^	0%
V43.2_25 °C_R_2 w	1.86 × 10^7^	0%

^1^ Resuscitated (R) strains were recovered from microcosms kept at 7, 15 and 25 °C, after 1–4 weeks (1 w–4 w) of entry into the VBNC state of the *Edwardsiella* original strain.

## Data Availability

Not applicable.
